# Reply to: Vitamin D: A single initial dose is not bogus if followed by an appropriate maintenance intake

**DOI:** 10.1002/jbm4.10605

**Published:** 2022-02-17

**Authors:** Richard B. Mazess, Heike A. Bischoff‐Ferrari, Bess Dawson‐Hughes

**Affiliations:** ^1^ Department of Medical Physics University of Wisconsin–Madison Madison WI USA; ^2^ Department of Geriatrics and Aging Research University of Zurich Zurich Switzerland; ^3^ City Hospital Zurich University Clinic for Aging Medicine Zurich Switzerland; ^4^ Jean Mayer US Department of Agriculture (USDA) Human Nutrition Research Center on Aging Tufts University Boston MA USA

## Peer Review

The peer review history for this article is available at https://publons.com/publon/10.1002/jbm4.10605.

**To The Editor**

We thank the reviewers for their comments on our review and agree that there is varied opinion on what constitutes bolus dosing of a medicament in terms of: (i) a single or multiple administration, (ii) the amount of the drug, and (iii) the intervals between administrations. Cholecalciferol boluses given at intervals in trials are often as large or larger than the equivalent daily dose (eg, 800 IU/day versus 100,000 IU at 1‐month intervals or perhaps 500,000 IU at 2‐month intervals). In contrast, a “loading dose” differs in being an initial dose of an agent several times higher than that given subsequently on a daily basis while the original loading dose is still partially present.

The reviewers correctly note that large intervals between cholecalciferol administration is ineffective. Research over the past 20 years led us to postulate that even a monthly bolus interval of cholecalciferol is ineffective for two reasons.^(^ ^1^, ^2^ ^)^

First, liver hydroxylation as well as cellular uptake give cholecalciferol a short 20‐hour half‐life minimizing its plasma level after a few days. The resultant 25‐hydroxyvitamin D (25(OH)D) level may remain elevated given its 15‐day half‐life but its metabolism to 1,25‐dihydroxyvitamin D (1,25‐(OH)2D) will be downregulated by the long‐lasting effect (months) of CYP24A1 encoding 24‐hydroxylase.

Second, the extrarenal cellular production of short‐lived (hours) immunological agents (cathelicidin [LL‐37], beta‐defensin, T regulatory cells [Tregs]) is dependent on the routine supply of absorbable cholecalciferol. The initial increases produced by a bolus disappear within 1–2 weeks even with elevated 25(OH)D, of which only about 0.05% is unbound and cellularly available. Hence cholecalciferol, usually circulating at more than 100 times the level of 25(OH)D, is more active for intracrine immune response. These factors make daily/weekly supplementation preferable for sustained immune response.^(^ ^3^ ^)^

Third, our review outlined a pattern across many outcomes where bolus doses were not effective, including acute respiratory infections, cancer mortality, and falls and fractures. The Murai study of coronavirus disease (COVID) patients using a single cholecalciferol bolus of 200,000 IU was one of these ineffective trials.^(^ ^4^ ^)^ In contrast, weekly dosing of COVID patients with 266 μg of calcifediol not only has been effective in two Spanish trials,^(^ ^5^ ^)^ but has been confirmed in clinical practice of that region. That calcifediol dose is the cholecalciferol equivalent of only 34,000 IU/week, and therefore unlikely to induce the downregulation of bolus dosing.

Finally, our review indicated that both circulatory 1,25(OH)2D level and 24‐hydroxylase enzyme activity are factors relevant to the effectiveness of vitamin D on immune system functioning, while the measured 25(OH)D level is not as important as many believe, as it may falsely signal efficacy after bolus dosing when both cholecalciferol is absent and 1,25(OH)2D is suppressed.

## Conflict of Interest

All authors state that they have no conflicts of interest.

## References

[1] Charoenngam N , Holick MF . Immunologic effects of vitamin D on human health and disease. Nutrients. 2020;12(7):2097. 10.3390/nu12072097 PMC7400911 32679784

[2] Bilezikian JP , Bikle D , Hewison M , et al. Mechanisms in endocrinology: vitamin D and COVID‐19. Eur J Endocrinol. 2020;183(5):R133‐R147. 32755992 10.1530/EJE-20-0665 PMC9494342

[3] Hollis BW , Wagner CL . Clinical review: the role of the parent compound vitamin D with respect to metabolism and function: why clinical dose intervals can affect clinical outcomes. J Clin Endocrinol Metab. 2013;98(12):4619‐4628. 24106283 10.1210/jc.2013-2653 PMC3849670

[4] Murai IH , Fernandes AL , Sales LP , et al. Effect of a single high dose of vitamin D3 on hospital length of stay in patients with moderate to severe COVID‐19: a randomized clinical trial. JAMA. 2021;325(11):1053‐1060. 33595634 10.1001/jama.2020.26848 PMC7890452

[5] Nogues X , Ovejero D , Pineda‐Moncusí M , et al. Calcifediol treatment and COVID‐19‐related outcomes. J Clin Endocrinol Metab. 2021;106(10):e4017‐e4027. 34097036 10.1210/clinem/dgab405 PMC8344647

